# The Spectrum of Podoplanin Expression in Encapsulating Peritoneal Sclerosis

**DOI:** 10.1371/journal.pone.0053382

**Published:** 2012-12-31

**Authors:** Niko Braun, M. Dominik Alscher, Peter Fritz, Joerg Latus, Ilka Edenhofer, Fabian Reimold, Seth L. Alper, Martin Kimmel, Dagmar Biegger, Maja Lindenmeyer, Clemens D. Cohen, Rudolf P. Wüthrich, Stephan Segerer

**Affiliations:** 1 Department of Internal Medicine, Division of General Internal Medicine and Nephrology, Robert-Bosch-Hospital, Stuttgart, Germany; 2 Institute of Digital Medicine, Stuttgart, Germany; 3 Department of Diagnostic Medicine, Division of Pathology, Robert-Bosch-Hospital, Stuttgart, Germany; 4 Division of Nephrology, University Hospital, Zurich, Switzerland; 5 Institute of Anatomy, University of Zurich, Zurich, Switzerland; 6 Division of Nephrology, Beth Israel Deaconess Medical Center, Department of Medicine, Harvard Medical School, Boston, United States of America; 7 Margarete Fischer-Bosch Institute of Clinical Pharmacology, University of Tuebingen, Stuttgart, Germany; 8 Institute of Physiology, University of Zurich, Zurich, Switzerland; Okayama University, Japan

## Abstract

Encapsulating peritoneal sclerosis (EPS) is a life threatening complication of peritoneal dialysis (PD). Podoplanin is a glycoprotein expressed by mesothelial cells, lymphatic endothelial cells, and myofibroblasts in peritoneal biopsies from patients with EPS. To evaluate podoplanin as a marker of EPS we measured podoplanin mRNA and described the morphological patterns of podoplanin-positive cells in EPS. Included were 20 peritoneal biopsies from patients with the diagnosis of EPS (n = 5), patients on PD without signs of EPS (n = 5), and control patients (uremic patients not on PD, n = 5, non-uremic patients n = 5). EPS patient biopsies revealed significantly elevated levels of podoplanin mRNA (p<0.05). In 24 peritoneal biopsies from patients with EPS, podoplanin and smooth muscle actin (SMA) were localized by immunohistochemistry. Four patterns of podoplanin distribution were distinguishable. The most common pattern (8 of 24) consisted of organized, longitudinal layers of podoplanin-positive cells and vessels in the fibrotic zone (“organized” pattern). 7 of 24 biopsies demonstrated a diffuse distribution of podoplanin-positive cells, accompanied by occasional, dense clusters of podoplanin-positive cells. Five biopsies exhibited a mixed pattern, with some diffuse areas and some organized areas ("mixed"). These contained cuboidal podoplanin-positive cells within SMA-negative epithelial structures embedded in extracellular matrix. Less frequently observed was the complete absence of, or only focal accumulations of podoplanin-positive fibroblasts outside of lymphatic vessels (podoplanin “low”, 4 of 24 biopsies). Patients in this group exhibited a lower index of systemic inflammation and a longer symptomatic period than in EPS patients with biopsies of the "mixed" type (p<0.05). In summary we confirm the increased expression of podoplanin in EPS, and distinguish EPS biopsies according to different podoplanin expression patterns which are associated with clinical parameters. Podoplanin might serve as a useful adjunct to the morphological workup of peritoneal biopsies.

## Introduction

Encapsulating peritoneal sclerosis (EPS) is a rare, but life-threatening complication of long-term PD [1], [2], [3]. Recent PD registries described rates of 0.7–3.3%, an incidence of 4.9 per 1000 person-years, and a mortality of 42% one year post diagnosis [4]. The diagnosis is based on the combination of clinical symptoms (bowel obstruction), radiological findings (suggesting extensive thickening of the peritoneal membrane as the cause of bowel obstruction), and/or the histo-morphological picture [1]. Peritoneal thickening, bowel tethering, peritoneal calcification, peritoneal enhancement and loculated fluid collections can be visualized by computed tomography [1]. Peritoneal biopsy histo-morphological features pathognomonic for EPS have not been defined, and the importance of peritoneal biopsy in the clinical diagnosis of EPS remains poorly established. Morphological signs such as mesothelial denudation, extreme fibrotic thickening, peritoneal fibroblast swelling, interstitial fibrosis, angiogenesis with increased capillary density, and mononuclear cell infiltration are all typical for EPS, but not specific [5], [6], [7]. Fibrin deposits may lead to adhesions and permanent scarring, eventually resulting in bowel obstruction.

Podoplanin, a member of a type-1 transmembrane sialomucin-like glycoprotein family, serves as a marker of lymphatic endothelial cells but is also expressed by mesothelial cells [8], [9]. In a previous study we described podoplanin expression in 69 peritoneal biopsies including 18 patients with EPS. 15 of these biopsies demonstrated a diffuse infiltration with podoplanin-positive cells [10]. These cells were identified as SMA-positive myofibroblasts, which did not express endothelial or other mesothelial markers [10]. This cell type was focally present in only 3 out of 16 specimens from PD patients without signs of EPS, and in none of 35 controls [10]. The accumulation of podoplanin-positive myofibroblasts in EPS was confirmed by Yaginuma and colleagues using immunoelectron microscopy [11].

Here we confirm the prominent expression of podoplanin using quantitative real-time RT-PCR, and describe four histological patterns of podoplanin-positive cells in EPS biopsies which, we propose, will facilitate morphologic diagnosis of EPS.

## Results

### Podoplanin mRNA Expression in Peritoneal Biopsies

**Figure 1:**
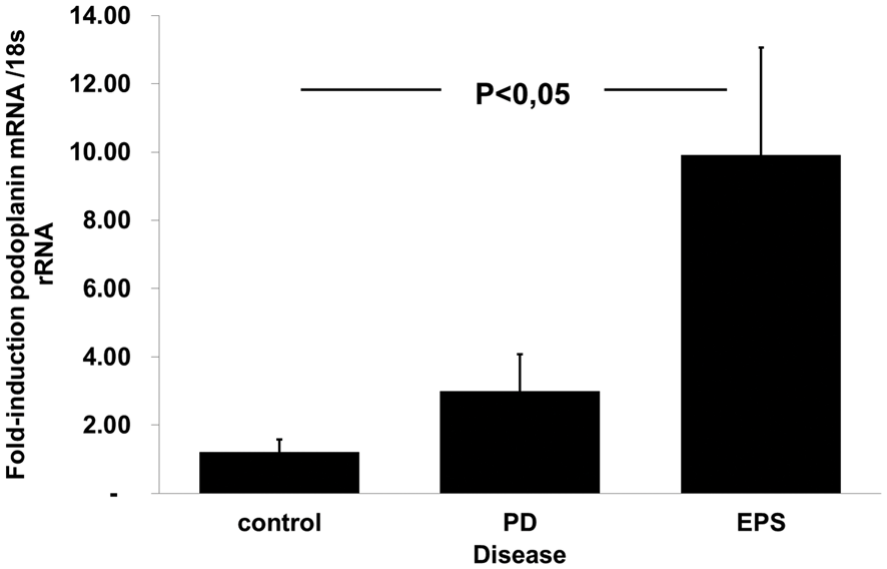
Podoplanin mRNA levels are increased in EPS. RNA samples from patients on PD without signs of EPS (n = 5), and with EPS (n = 5), as well as control biopsies from patients with uremia (n = 5) and normal peritoneum taken during laparatomy (n = 5). The two groups were pooled as "control" samples. Podoplanin mRNA levels were analyzed by real-time RT-PCR. The mean fold induction of podoplanin mRNA was normalized to normal control samples.

To evaluate podoplanin expression on transcript level we performed real-time RT-PCR on peritoneal biopsies (Table 1) taken from uremic patients not on PD (n = 5), patients on PD (n = 5), and from PD patients with clinical signs of EPS (n = 5). An additional set of control biopsies were taken from normal peritoneum during abdominal surgery (see material and methods). Both control groups were pooled in this analysis (Figure 1). A prominent and statistically significant induction of podoplanin mRNA was demonstrated. Therefore the previously described accumulation of podoplanin-positive myofibroblasts in EPS was associated with significant induction of podoplanin mRNA expression [10], [11].

**Table 1:** Clinical information and laboratory values of patients from whom originated biopsy specimens studied by RT-PCR.

Variable	Uremic patients(not on PD)	PD	EPS	Normal biopsies
n =	5	5	5	5
Gender (male:female)	3∶2	4∶1	4∶1	1∶4
Age (years;mean ±SD)	54.4 (±16.2)	64.4 (±11.8)	51.6 (±11.0)	56.2 (±12.3)
PD-duration in months		35.2 (±38.2), n.s.	72.6 (±24.3)	
Peritonitis		1∶22 months	1∶46 months	
PDF: Neutral		4	2	
Acidic		0	2	
N.D.		1	1	
**Transporter status**				
High/high average		2	3	
Low/low average		1	0	
N.D.		1	2	
Icodextrin		2/5	4/5	
Diabetes	4/5	2/5	0/5	0/5
Smoker	2/5	1/5	2/5	0/5
Hypertension	3/5	3/5	5/5	2/5
Hb (g/dl ± SD [13]–[18])	10.7 (±1.0)	12.2 (±1.9)	9.7 (±1.8)	12.4 (±2.6)
Leukocytes (G/L ± SD [4.0–11.3])	8.5 (±1.5)	6.4 (±1.4)	6.7 (±3.5)	5.4 (±1.3)
CRP (mg/dl ± SD [<0.1])	1.1 (±2.4)	1.9 (±2.4)	6.4 (±7.5)	0.1 (±0.1)
Phosphate (mmol/l [0.68–1.68])	1.7 (±0.25)	1.3 (±0.3)	1.2 (±2.2)	
Calcium (mmol/l [1.90–2.70])	2.05 (±0.12)	2.2 (±0.1)	2.1 (±0.3)	2.28 (±0.1)
PTH (pmol/l [1.1–7.3])	34.3 (±6.8)	31.1 (±26)	46 (±49.4)	
Urea-N (mg/dl [10–25])	62.2 (±22.9)	42.5 (±22.2)	35.4 (±14.2)	
Creatinine (mg/dl [0.5–1.4])	5.1 (±0.94)	5.1 (±2.3)	6.4 (±2.9)	0.8 (±0.14)

PD, peritoneal dialysis; EPS, encapsulating peritoneal sclerosis; n.s. not significant compared to EPS. PDF, peritoneal dialysis fluid; Hb, haemoglobin; PTH, parathyroid hormone; CRP, C-reactive protein.

### Podoplanin Patterns by Immunohistochemistry

The clinical and laboratory data of the 24 patients with EPS included in the morphological analysis are presented in Table 2. Podoplanin staining was first analyzed without knowledge of the clinical information. This analysis led to segregation into four groups of podoplanin patterns. Podoplanin-positive lymphatic vessels were present in all four groups, whereas the groups differed in the appearance of podoplanin-positive cells with the morphology of myofibroblasts.

**Table 2:** Clinical information and laboratory values of patients with EPS.

Variable		EPS
**n = **		24
**Gender (male:female)**		21/3
**Age (years; mean ±SD)**		55.1 (±11.0)
**PD-duration (months)**		80 (±35)
**Peritonitis episodes**		60 in 1919 months (1∶32)
**PDF**		
**Neutral**		9/24
**Acidic**		8/24
**Both or N.D.**		7/24
**Transporter status**		11
**High/high average**		5
**Low/low average**		8
**N.D. last 6 months**		
**Icodextrin**		18/24
**Diabetes**		17/24
**Smoker**		9/24
**Hypertension**		22/24
**Hb (g/dl, 13–18)**		10.6 (±2.9)
**Leukocytes (G/L, 4.0–11.3)**		8.7 (±3.4)
**Phosphate (mmol/l, 0.68–1.68)**		1.3 (±0.5)
**Calcium (mmol/l, 1.9–2.7)**		2.3 (±0.3)
**PTH (pmol/l, 1.1–7.3)**		25.6 (±25.1)
**Urea-N (mg/dl, 10–25)**		41.2 (±15.6)
**Creatinine (mg/dl, 0.5–1.4)**		6.9 (±2.2)
**Time of onset of complaints to Surgery (months)**		7.1 (±5.5)
**CRP (mg/dl, <0.1)**		9.0 (±10.7)

Hb, haemoglobin; EPS, encapsulating peritoneal sclerosis; PD, peritoneal dialysis;

PDF, peritoneal dialysis fluid; PTH, parathyroid hormone; CRP, C-reactive protein; SD, standard deviation.

**Figure 2:**
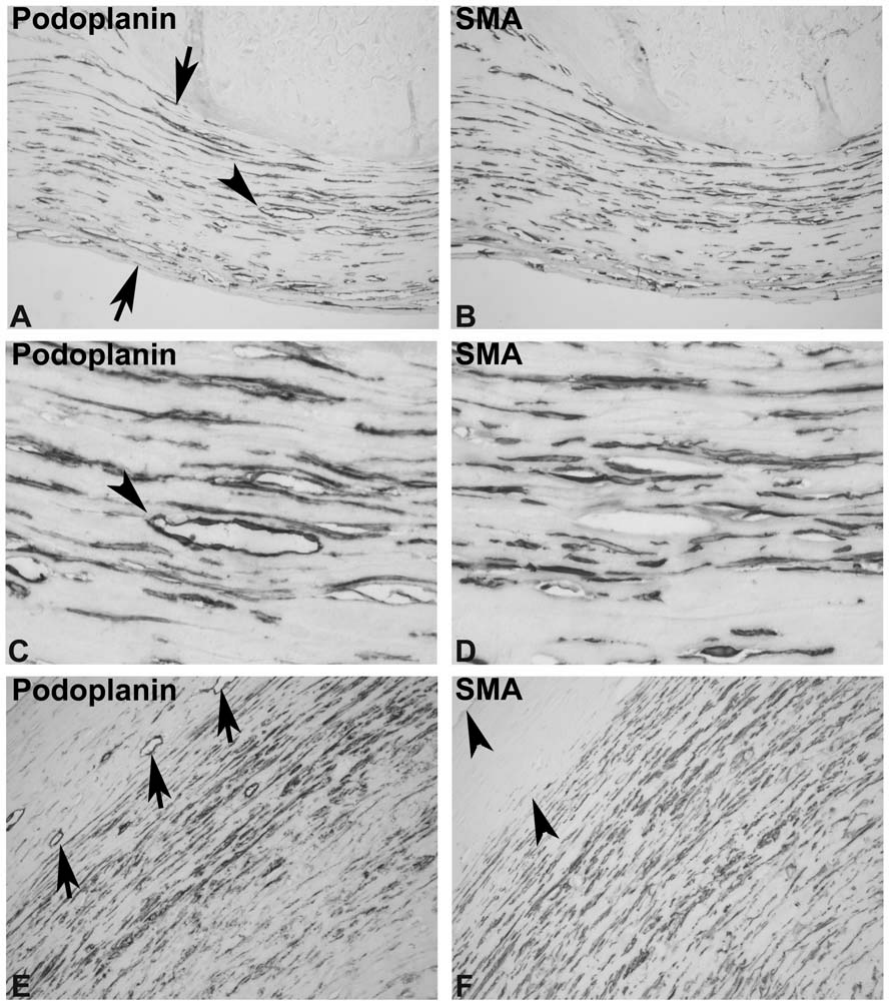
Examples of the "organized" pattern of podoplanin in EPS. Peritoneal biopsies from patients with EPS were stained with monoclonal antibodies against podoplanin (A, C, E). Consecutive section stained with monoclonal antibody against SMA (B, D, F) showed that many cells appeared to express both proteins. In the fibrotic zone (between arrows in A) longitudinal layers of podoplanin-positive cells and vessels (A, C arrowhead) are present, and on consecutive sections SMA-positive cells are detected. These “organized” longitudinal layers led to the description as an "organized" pattern. The superficial layer illustrated in E (upper left corner) contains only some podoplanin-positive vessels, but few SMA-positive cells. Below this superficial layer a prominent zone with podoplanin- and SMA-positive cells is present, as in panels A and B. Panels A-D are from a single individual, panels E and F are from a different individual. (Original magnification, 200X in A, B, E, F; 630X in C, D.).

The most common pattern of podoplanin-positive cells was a prominent staining of the superficial fibrotic layer, with longitudinal alignment of podoplanin-positive cells and vessels (Figure 2 A). These longitudinal layers appeared well organized (Figure 2 A, E) with podoplanin-positive cells and vessels arrayed in parallel orientation (“organized” pattern, 8 out 24 biopsies). The corresponding distribution of SMA-positive cells was similar to the pattern of podoplanin (Figure 2 B, D). In the podoplanin-stained sections, prominent lymphatic vessels present in the fibrotic layer (Figure 2 A, B arrowheads) were negative for SMA expression on consecutive sections (Figure 2 D). In some fibrotic zones the superficial layer contained fewer podoplanin- and SMA-positive cells (Figure 2 F left upper corner, between arrowheads). Podoplanin-positive lymphatic vessels were detectable in this superficial layer (arrows in Figure 2 E).

**Figure 3:**
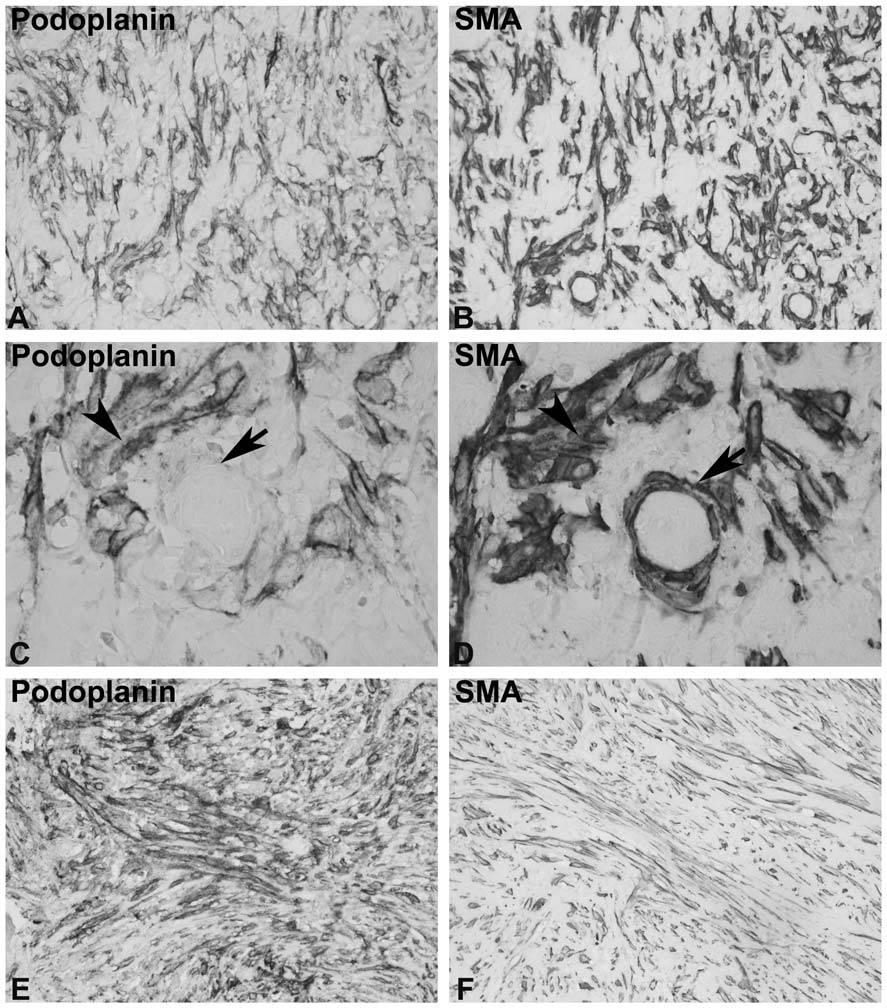
Examples of the "diffuse" pattern. Peritoneal biopsies from patients with EPS were stained with monoclonal antibodies against podoplanin (A, C, E). Consecutive sections were stained with a monoclonal antibody against SMA (B, D, F). The diffusely distributed and randomly oriented pattern of individual podoplanin-positive cells separated by matrix is illustrated in A, and at higher magnification in C. In E the same pattern is illustrated with more densely distributed podoplanin-positive cells. At a higher magnification (C, D) the SMA-positive cells of arteries (arrow) were podoplanin-negative, but the SMA-positive myofibroblasts (arrowhead) were podoplanin-positive. Panels A-D are from a single individual, panels E and F are from a different individual. (Original magnification, 200X in A, B, E, F; 630X in C, D.).

The second most common appearance of podoplanin (7 out of 24 biopsies) was in a diffuse pattern with an irregular, random distribution of podoplanin-positive cells (“diffuse” pattern, Figure 3). The cells seemed to be randomly distributed and oriented within the fibrotic zones (Figure 3 A), in contrast to the longitudinal orientation in the "organized" pattern (Figure 2 A). Podoplanin-positive cells occupied a larger proportion of the visual fields in biopsies of "diffuse" pattern (Figure 3 E). The individual podoplanin-positive cells were embedded in extracellular matrix (Figure 3 A). Other areas demonstrated dense accumulations of podoplanin-positive cells with correspondingly less prominent extracellular matrix (Figure 3 E). The SMA expression pattern was very similar to the podoplanin staining (Figure 3 B, D, F), but SMA-positive smooth muscle cells in vessel walls were podoplanin-negative (Figure 3 C, D).

**Figure 4:**
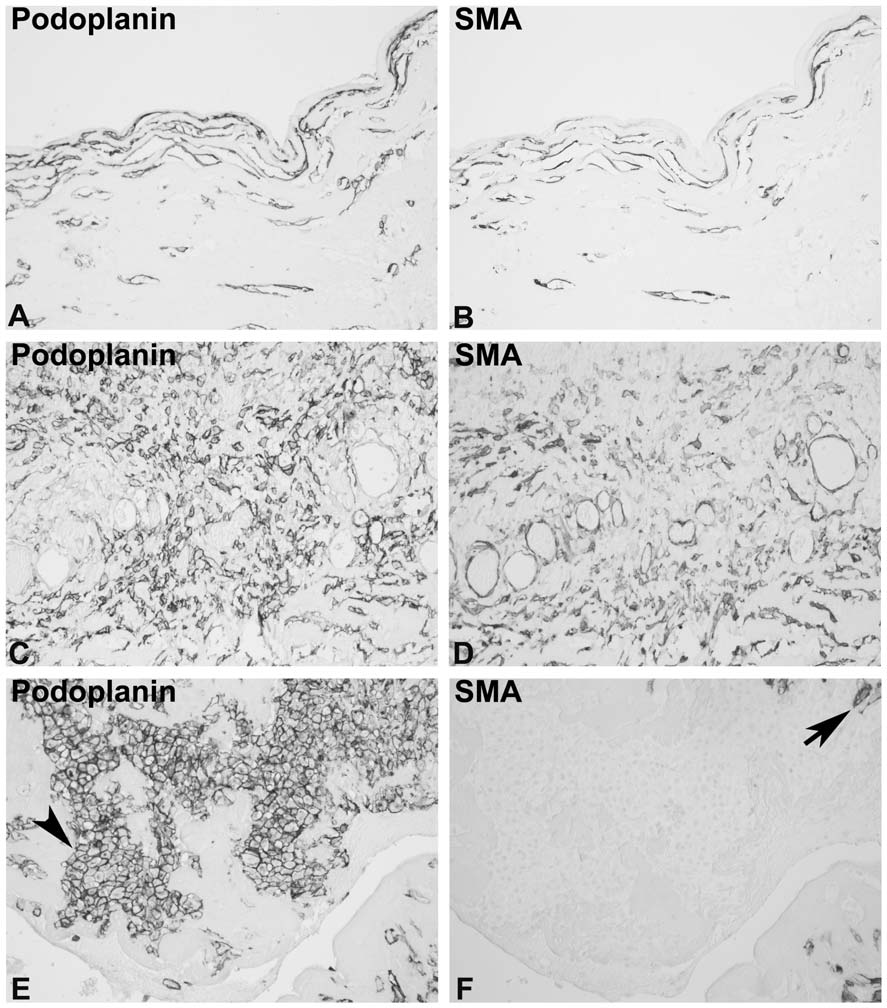
Illustration of the “mixed” pattern. Peritoneal biopsies from patients with EPS were stained with monoclonal antibodies against podoplanin (A, C, E). Consecutive sections were stained with monoclonal antibody against SMA (B, D, F). Examples of “organized” (A, B) and “diffuse” (C, D) patterns are illustrated from the same tissue specimen. An area of podoplanin-positive, cuboidal cells embedded in matrix is illustrated in E (arrowhead), but these cells were SMA negative on consecutive sections (F). An SMA-positive myofibroblast is illustrated by the arrow (F, upper right). Panels A-D are from a single individual, panels E and F are from a different individual. (Original magnification, 200X.).

In 5 out of 24 biopsies areas of both “organized” (Figure 4 A, B) and “diffuse” pattern (Figure 4 C, D) were observed, which we referred to as “mixed” pattern. These biopsies demonstrated the most variable appearance of podoplanin-positive cells. Interestingly, these biopsies contained areas of cuboidal podoplanin-positive cells, which were embedded in extracellular matrix (Figure 4 E, F). Consecutive sections revealed that these cells (arrowhead in 4 E) were SMA-negative (Figure 4 F). It is important to note that this podoplanin-positive, SMA-negative cell type differs in morphology (cuboidal rather than fibroblastic) and in SMA expression from the majority of podoplanin-positive cells. Clusters of podoplanin-positive, SMA-negative cuboidal cells were completely surrounded by dense extracellular matrix. Some podoplanin positive cells were separated from these structures. Also these cells were completely surrounded by extracellular matrix on consecutive sections a major part (but not all) demonstrated expression of calretinin (indicating an mesothelial origin/and or mesothelial differentiation, not illustrated).

**Figure 5:**
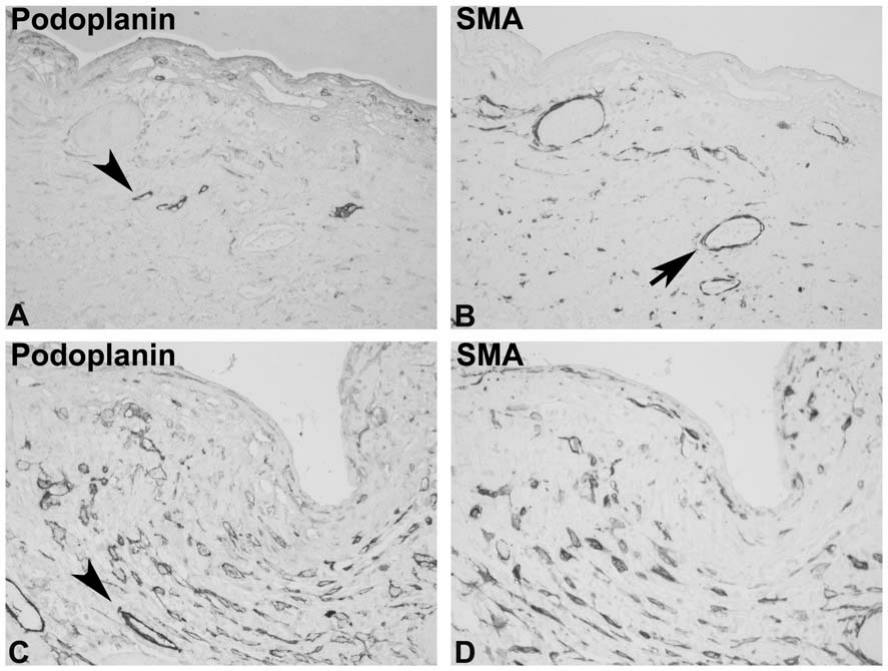
Example of the podoplanin “low” pattern. A peritoneal biopsy from an EPS patient was stained with monoclonal antibodies against podoplanin (A, C), and consecutive sections were stained with a monoclonal antibody against SMA (B, D, orig. X200). Panel A illustrates an area in which the fibrotic zone does not demonstrate a significant accumulation of podoplanin-positive cells (the arrowhead show some positive small vessels). In small areas of the biopsy the typical presence of podoplanin-positive myofibroblast was detectable The arrowhead in B marks a podoplanin-positive lymphatic vessel, not stained by SMA on the consecutive section (D).

In four out of 24 biopsies the expression of podoplanin was mainly restricted to lymphatic endothelial cells (Figure 5). Particularly in the fibrotic zones, where a prominent accumulation of podoplanin-positive cells was present in the biopsies with the other patterns, no podoplanin-positive myofibroblasts were detectable (podoplanin “low” pattern. Figure 5 A, B). In some biopsies focal accumulations of podoplanin-positive fibroblastic cells were detected (Figure 5 C, D). In this histological class of biopsies, location and detection of these scarce, focal areas of podoplanin-positive cells may require analysis of several biopsy specimens from one individual.

**Figure 6:**
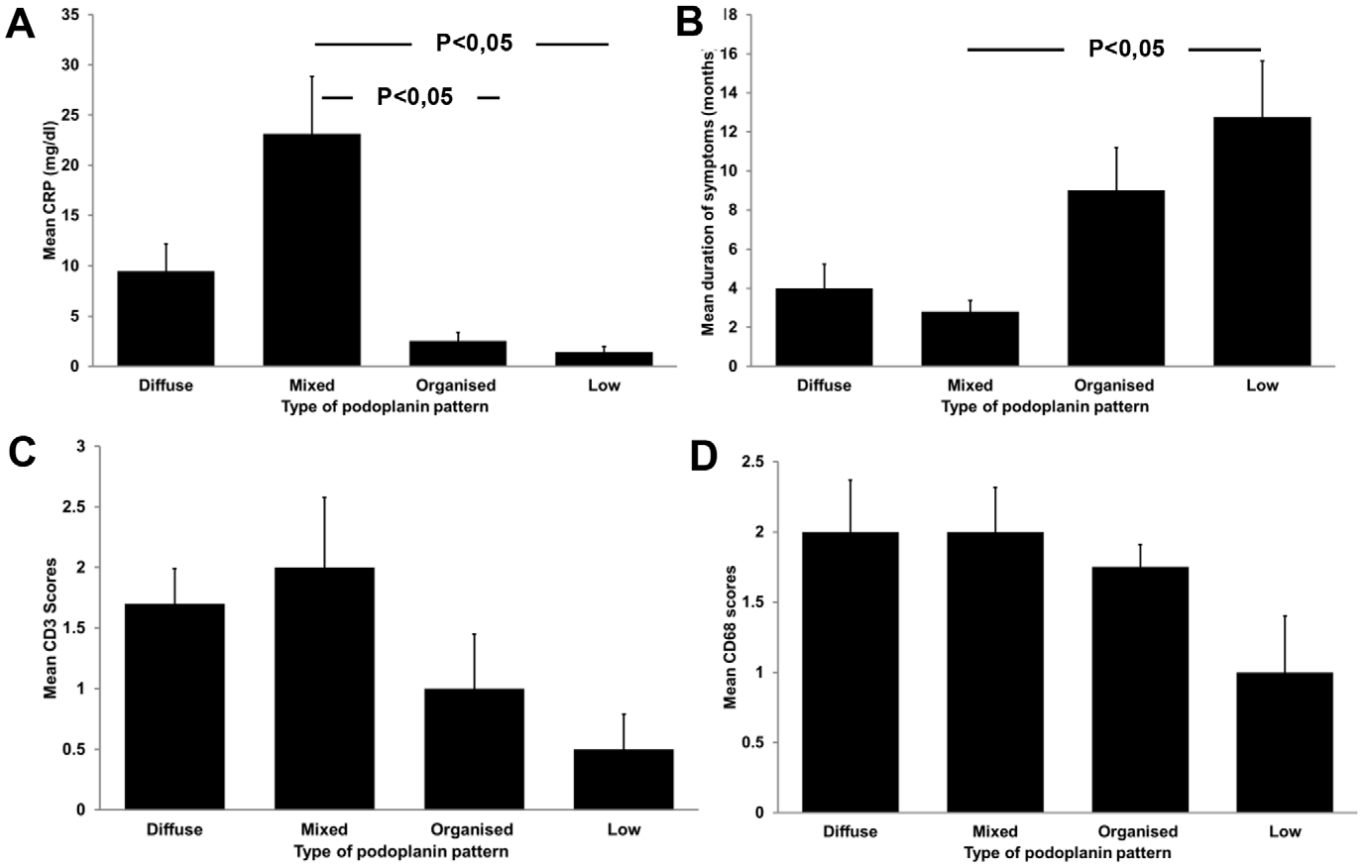
Association between podoplanin pattern and clinical parameters and morphological scores of inflammatory cells. Illustrated are the mean C reactive protein (CRP) levels (A), the mean duration of symptoms (in months, B), the mean scores for CD3 positive T cells (C) and the mean scores for CD68 positive cells (D) for the four histological groups of podoplanin patterns. The infiltrating cells were scored semi-quantitatively as described in materials and methods.

Importantly, patients with biopsies with the podoplanin “low” pattern had a significantly lower level of systemic inflammation (as reflected by serum C-reactive protein concentrations, Figure 6 A), but the longest time with symptoms (Figure 6 B). The groups did not differ in mean age, time on PD, or number or frequency of peritonitis episodes (Table 3). The number of CD20 positive B cells, CD3 T cells and CD68 positive macrophages/DCs were scored semi-quantitatively. Most biopsies demonstrated either a mild (score 1) or severe (score 2) diffuse infiltration of CD3 and more prominent CD68 positive cells. CD20 positive B cells were rare within the fibrotic membranes. A single biopsy contained two nodular accumulations on the abluminal side of the fibrotic membrane. Larger accumualtions of infiltrating cells (score 3) were rare. The mean scores of CD3 and CD68 positive infiltrating cells in the biopsies were similarly distributed as the C-reactive protein, with the highest scores in biopsies with a diffuse or mixed pattern (Figure 6 C, D). B cells demonstrated low scores and no differences between the groups.

**Table 3:** Clinical information and laboratory values according to the morphological pattern of podoplanin.

	Diffuse	Mixed	Organised	Low
**n = **	7	5	8	4
**Gender (male:female)**	7/0	4/1	7/1	4/0
**Age (years;mean ±SD)**	51.7 (±15.9)	46.4 (±7.3)	55.5 (±12.2)	47.3 (±17.2)
**PD-duration in months**	71 (±37)	99 (±33)	86 (±29)	59 (±39)
**Peritonitis**	1∶26	1∶37	1∶33	1∶26
**PDF**				
**Neutral**	3	1	4	1
**Acidic**	2	1	3	2
**Both or N.D.**	2	3	1	1
**Transporter status**				
**High/high average**	2	3	5	1
**Low/low average**	1	1	1	2
**N.D.**	4	1	2	1
**Icodextrin**	4	5	6	3
**Diabetes**	2	1	2	1
**Smoker**	3	2	2	2
**Hypertension**	6	4	8	4
**Hb (g/dl ± SD ** [13]–[18] **)**	84 (±31.1)	115 (±29.3)	116 (±21.0)	113 (±26.4)
**Leukocytes (G/L ±SD [4.0–11.3])**	8.4 (±2.3)	11.2 (±5.2)	8.6 (±3)	6.5 (±2.2)
**Phosphate (mmol/l [0.68–1.68])**	1.7 (±0.6)	1.0 (±0.5)	1.3 (±0.5)	1.4 (±0.6)
**Calcium (mmol/l [1.90–2.70])**	2.1 (±0.2)	2.3 (±0.3)	2.4 (±0.3)	2.3 (±0.4)
**PTH (pmol/l [1.1–7.3])**	28.5 (±17.6)	12.9 (±8.8)	21 (±30.5)	43 (±30.8)
**Urea-N (mg/dl [10–25])**	92.1 (±32)	69.8 (±27.5)	87.4 (±27.8)	105.8 (±52.3)
**Creatinine (mg/dl [0.5–1.4])**	6.8 (±2.4)	6.8 (±2.1)	7 (±2.2)	6.7 (±3.1)

PD, peritoneal dialysis; EPS, encapsulating peritoneal sclerosis; PDF, peritoneal dialysis fluid; Hb, haemoglobin; PTH, parathyroid hormone.

## Discussion

In a previous study we described a podoplanin-positive, SMA positive cell population in 15 of 18 biopsies from patients with EPS, whereas only focal accumulations of podoplanin-positive cells were present in biopsies from 3 of 16 patients on PD without signs of EPS [10]. We thus suggested that podoplanin staining might be suitable as an adjunct to the morphological diagnosis of EPS. The current study had two goals. The first was to examine podoplanin expression in EPS with a different technique (i.e. mRNA measurement by quantitative real-time RT-PCR). The second goal was to further describe the pattern of podoplanin-positive cells, particularly in those biopsies without the typical accumulation of podoplanin-positive myofibroblasts.

Using quantitative real-time RT-PCR we were able to confirm increased levels of podoplanin mRNA in peritoneal biopsies from patients with EPS on PD. Furthermore, a recent study confirmed the existence of these podoplanin-positive fibroblastic cells by both immunohistochemistry and by immunoelectron microscopy [11]. Thus, the first goal of the paper was achieved, as another technique demonstrated increased podoplanin expression in a different group of EPS patients.

The second part of the work aimed to extend the histological description and pattern(s) of podoplanin-positive cells in peritoneal biopsies. We found that the biopsies could be separated into four morphological groups. From the diagnostic point of view, the group with rare podoplanin-positive cells (“podoplanin low”) is an important one, as this category might generate false negative results for EPS. Four of 24 patients demonstrated podoplanin-positive vessels (lymphatics), but only small and focal sites of accumulation of podoplanin-positive cells. In our previous study, the proportion of patients with this pattern was similar (with 3 of 16). Therefore, between 15 and 20% of biopsies from patients with EPS exhibit a “low” podoplanin pattern. Patients with this histological pattern had the lowest level of systemic inflammation (as judged by serum levels of C-reactive protein), and the longest history of symptoms. Therefore, this pattern could reflect a late (and/or slowly progressive) disease state (fibrotic, or “burnt out”). It is important to note that these small clusters of podoplanin-positive cells can usually be detected when sufficient material is available and thoroughly examined, but these areas can easily be missed on smaller biopsies.

Two additional, distinct patterns and a mixed pattern could be identified. The "organized" pattern and the "diffuse" pattern were observed with equal frequency, and were together most commonly found. The "organized' pattern was characterized by a longitudinal organization of podoplanin-positive cells throughout the fibrotic layer or the basal part of the fibrotic zone. In contrast, the "diffuse" pattern was distinguished by a random accumulation of podoplanin-positive cells across a larger area of biopsy. Five of 24 biopsies demonstrated a "mixed" pattern (with features of both "organized" and "diffuse" patterns). The “mixed” pattern group exhibited the highest level of inflammation and the shortest history of symptoms prior to surgery. The "mixed" and the "diffuse" types likely reflect the most active and aggressive phases of EPS, with sites of very prominent podoplanin- and SMA-positive cell accumulation. Furthermore the “mixed” and the “diffuse” type demonstrated the strongest infiltration by T cells and CD68 positive macrophages/DCs.

The morphological patterns were not associated with the number of peritonitis episodes and the groups did not differ in age, time on PD, Icodextrin exposure, parathyroid hormon, and calcium. Also not quite significant, patients with mixed pattern demonstrated a trend towards higher leukocytes, lower urea and lower phosphate (likely reflecting poorer nutritional status).

It is currently unclear whether the different morphological podoplanin patterns are related to differences in the quantitative podoplanin mRNA expression. Particularly, the podoplanin “low” pattern is likely associated with a decreased podoplanin mRNA expression. Currently, the materials available for matched podoplanin staining and mRNA quantification were not sufficient to answer this question, but it will be evaluated in a future study.

The fibroblastic podoplanin- and SMA-positive cell type typical for EPS was found to be negative for calretinin (a marker of mesothelial cells [10]. Podoplanin positive stromal cells (called lymphoid stromal cells,) have been described as follicular reticular cells (also positive for SMA) in T cell zones of secondary lymphatic organs, in thymic medulla, in intestinal lamina propria, and in tertiary lymphoid organs formed during chronic infiltration [17], [18], [19]. During development of secondary lymphoid organs the lymphoid tissue inducer cells activate podoplanin positive stromal cells via lymphotoxin to release chemokines and upregulate adhesion molecules [18]. Some forms of Inflammation recapitulate the formation of lymphoid stromal cells as the accumulation of podoplanin positive stromal cells was demonstrated in different inflammatory models in the mouse (e.g. models of autoimmunity, inflammation of mouse ears induced by adjuvant) [18]. During inflammation the lymphoid stromal cells resulted from local proliferation of non-epithelial precursor cells [18]. The development of these cells seems to be dependent on the injury process. Studies in chronically inflamed kidneys did not demonstrate the accumulation of podoplanin positive myofibroblasts, whereas SMA positive cells form a prominent part of the interstitial fibroblasts in chronic renal injury [16]. Furthermore, in patients on PD with simple peritoneal fibrosis, diffuse accumulation of podoplanin positive myofibroblasts was rarely detected. Therefore the podoplanin positive cells in EPS reflect features of lymphoid stromal cells. As nodular infiltrates were rarely present full tertiary lymphoid organs were not present, these cells do not seem to promote the formation of lymphoid tissue in the EPS membranes. Future studies will need to further describe these cells with other markers of lymphoid stromal cells. The question remains whether these cells are the consequence of the injury process of EPS or a driving force.

Another podoplanin positive cell type has been described in mice. During zymosan peritonitis a F4/80 positive cell expressing podoplanin has been described and called fibroblastic macrophages [20]. In our study the overall pattern of CD68 (a marker of human monocyte/macrophages/DCs) did not match the podoplanin staining, but further studies using double labelling need to evaluate whether similar fibroblastic macrophages are present in human EPS.

In the biopsies with a “mixed” pattern clusters of podoplanin-positive cuboidal cells were found embedded in extracellular-matrix (illustrated in Figure 4E). As these cells cells were SMA negative, these might reflect mesothelial cells [21]. In parallel staining for calretinin (as a marker of mesothelial cells) a major part of these cells demonstrated calretinin expression, but a smaller part did not. Therefore these cells do not seem to be typical mesothelial cells but the differentiation of these cells need further description. In Figure 4C the excess of podoplanin-positive cells over SMA-positive cells (in Figure 4 D) may reflect the presence of this cell type. These areas might reflect sites of early epithelial-to-mesenchymal cell transition or a cell on the way towards a mesothelial phenotype.

The question remains whether the patterns illustrated reflect a continuum (likely from "mixed/diffuse" pattern via "organized" towards "low" pattern) or different disease entities. The clinical data suggest that the "mixed/diffuse" pattern reflects earlier (active) phases, whereas the "organized" and, particularly, the podoplanin “low” pattern are rather later stages. If true, then the “low” pattern might not be susceptible to anti-inflammatory treatment, a hypothesis that could be tested. This novel stratification of EPS patients into groups exhibiting distinct podoplanin expression patterns could be of significant diagnostic and prognostic impact, if it can be confirmed in other EPS biopsy registries.

## Methods

All peritoneal biopsies were obtained from the peritoneal biopsy registry at the Robert-Bosch-Hospital, Stuttgart, Germany. The human peritoneal tissue, blood and peritoneal dialysate for research purposes were collected after written consent of the patient was given. The study was approved by the local ethics committee (#322/2009BO1, Eberhard-Karls University Tuebingen, Germany).

Additionally, shortly after tissue excision, 20 samples from patients on PD without EPS (n = 5), patients with EPS (n = 5), uremic patients not on PD (n = 5), and normal tissue taken during cholecystectomy (n = 3), hemicolectomy (n = 1) and closure of loop-colostomy (n = 1), all without signs of systemic and local inflammation, were washed in 0.9% saline solution, placed in RNAlater (Qiagen, Hilden, Germany), then stored at −80°C for subsequent RNA extraction.

Clinical data collection included demographic data, cause of primary renal disease, comorbidities (diabetes, hypertension and smoking status), PD details and the date of dialysis initiation. Body mass index, peritonitis rate, medications. and time of onset of symptoms were also recorded. The diagnosis of EPS was made according to the clinical criteria of Nakamoto et al. [12], the radiological criteria of Vlijm et al. [13] and the histological criteria of Honda et al. [5]. Biopsies of the parietal peritoneum were taken, formalin-fixed and embedded in paraffin following routine protocols. All patients were on hemodialysis after surgery.

### Quantitative Real-time RT-PCR

RNA was isolated from tissues frozen and immersed in RNAlater (Qiagen), using the miRNeasy Mini-Kit (Qiagen) according to the description of the manufacturer. 50–100 mg tissue samples were incubated in 0.7 ml Qiazol reagent and homogenized using a rotor-stator homogenizer (Ultra-Turrax T8, IKA-Werke Staufen, Germany) for 1 minute. The homogenate was extracted with 0.14 ml chloroform, and phase separation of the solution was achieved by centrifugation. The clear, aqueous supernatant containing total RNA was removed, and total RNA was twice precipitated with 75% ethanol, and re-suspended in nuclease-free water.

RNA was measured using a NanoDrop Spectrophotometer 2000c (Peqlab Biotechnologie GmbH, Erlangen, Germany). RNA integrity was assessed using the Agilent 2100 Bioanalyzer (Agilent Technologies, Santa Clara, CA). First-strand cDNA was synthesized with TaqMan RT reagents (Applied Biosystems, Darmstadt, Germany). Pre-developed TaqMan reagents were used for human podoplanin and housekeeping genes GAPDH and 18SrRNA (Applied Biosystems, Darmstadt, Germany). The mRNA expression was analyzed by the delta delta Ct method as previously described [14].

### Immunohistochemistry

Immunohistochemistry was performed as previously described [10], [15]. In brief, paraffin-embedded tissue sections were deparaffinized in xylene, rehydrated in a graded series of ethanols, and incubated in 3% hydrogen peroxide (to block endogenous peroxidases). Antigen retrieval was performed in an autoclave oven, using antigen retrieval solution (Vector, Burlingame, CA). The primary antibodies were applied for 1 hour. Incubation with biotinylated secondary reagents (Vector) was performed for 30 minutes, followed by washing, then exposure to ABC reagent (Vector). 3′3′diaminobenzidine (DAB, Sigma, Taufkirchen, Germany) with metal enhancement (resulting in a black colour product) was used as a detection system. Nuclei were counterstained with methyl green.

A monoclonal mouse anti-human podoplanin antibody (D2–40, Signet Laboratories, Dedham, MA) was used on all biopsies [15], [16]. As control tissues we used sections of human renal allograft nephrectomies, including the replacement of the primary antibody by isotype-matched control immunoglobulins (Figure S1). These controls did not demonstrate positive staining (Figure S1 B). For detection of SMA a monoclonal mouse antibody was used (1A4, DakoCytomation, Glostrup, Denmark), which stains smooth muscle cells in arterial walls and in myofibroblasts (Figure S1 C, D). Additional sections were stained with a monoclonal antibody against CD68 (Clone PG-M1, DAKO Germany, Hamburg), with a monoclonal antibody against CD3 (clone: CD3–12, rat anti-human, Serotec, Oxford, UK), and with a monoclonal antibody against CD20 (clone L26; DakoCytomation, Dako Deutschland, Hamburg, Germany) [16]. Selected biopsies were stained with calretinin (Dak Calret 1, DakoCytomation, Glostrup, Denmark) a marker of mesothelial cells. The extent of inflammatory infiltrates were semi-quantitatively scored from 0 (no or scattered cells), 1 (milde diffuse infiltrates), 2 (severe diffuse infiltrates) to 3 (severe diffuse infiltrates with larger cell accumulations) by an observer blinded to the morphological podoplanin pattern and the clinical information.

### Statistical Analysis

Statistical analysis was performed using InStat® software (Version 3.05, Intuitive Software for Science, San Diego, CA). For comparison of means, the non-parametric Kruskal-Wallis test and Dunn's multiple comparisons test were applied. A p<0.05 was considered to be significant. Error bars demonstrate standard error of the mean (SEM).

### Conclusions

The current study confirms elevated podoplanin expression in EPS peritoneal biopsies. The morphological evaluation of podoplanin can separate histological groups with different clinical features, and in the future might guide both diagnosis and treatment of EPS. The similarity of these podoplanin positive cells with lymphoid stromal cells needs further evaluation.

## Supporting Information

Figure S1 **Illustration of podoplanin and SMA in control tissue.** Immunohistochemistry was performed on tissue sections from an allograft nephrectomy (A, C), with monoclonal antibodies against podoplanin (A) and smooth muscle actin (C). Consecutive sections of the renal allograft were stained with the isotype immunoglobulin control (as negative control B, D). Note the staining of periarterial lymphatic vessels (arrowhead in A) and the absence of staining in B. Panel C shows SMA-positive cells in the walls of an artery (arrowhead) and an arteriole (arrow). No staining is present in the isotype immunoglobulin control (D). (Original magnification, 200X)
(TIF)
