# Supplementary figures and images for: The Spectrum of Podoplanin Expression in Encapsulating Peritoneal Sclerosis
Source: PLoS One. 2012 Dec 31;7(12):e53382. doi: 10.1371/journal.pone.0053382 (PMC3534056; doi:10.1371/journal.pone.0053382)

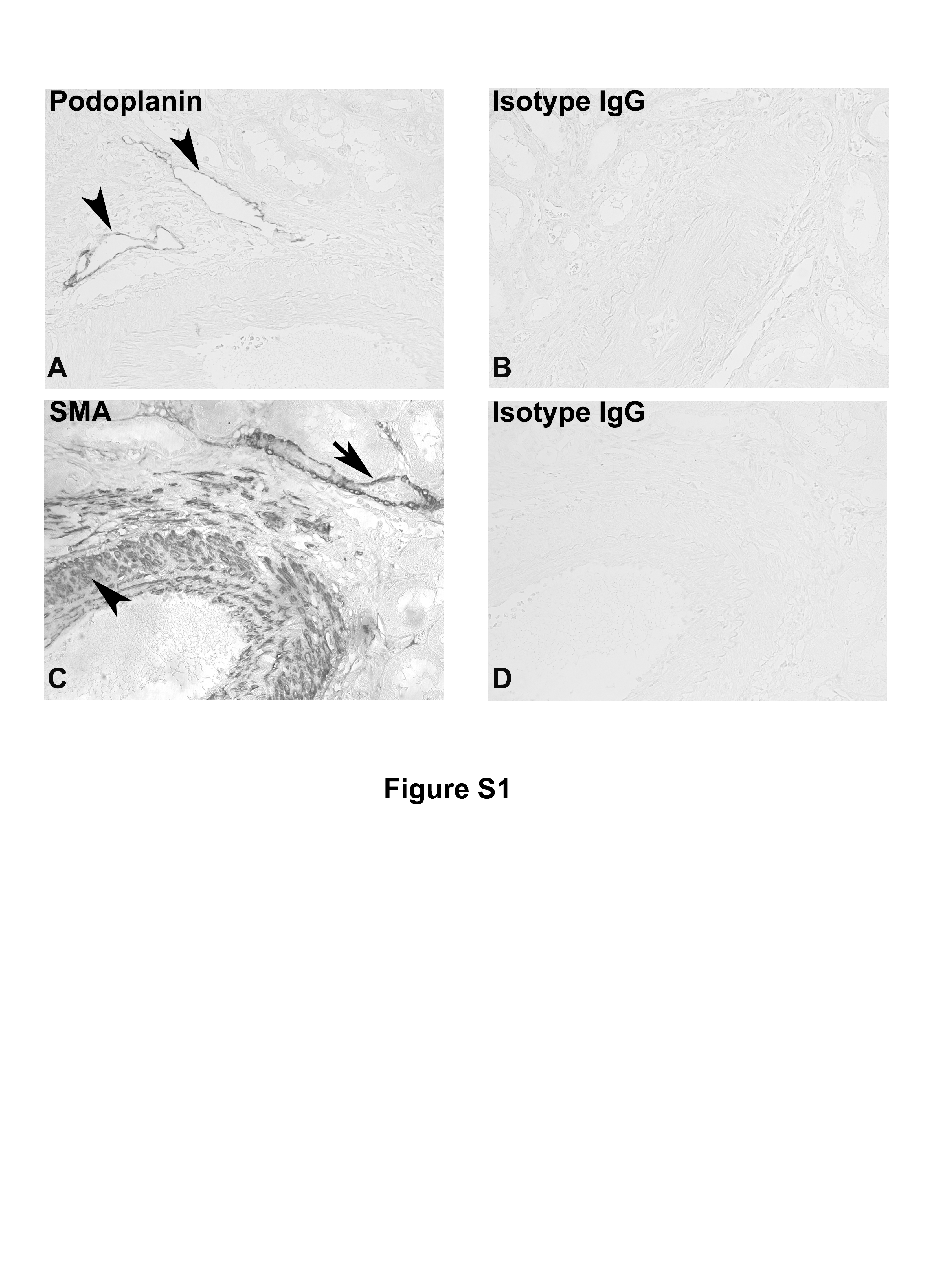

Supplement: Figure S1 — Illustration of podoplanin and SMA in control tissue. Immunohistochemistry was performed on tissue sections from an allograft nephrectomy (A, C), with monoclonal antibodies against podoplanin (A) and smooth muscle actin (C). Consecutive sections of the renal allograft were stained with the isotype immunoglobulin control (as negative control B, D). Note the staining of periarterial lymphatic vessels (arrowhead in A) and the absence of staining in B. Panel C shows SMA-positive cells in the walls of an artery (arrowhead) and an arteriole (arrow). No staining is present in the isotype immunoglobulin control (D). (Original magnification, 200X) (TIF) [file pone.0053382.s001.tif]
